# A Survey on Wearable Sensors for Mental Health Monitoring

**DOI:** 10.3390/s23031330

**Published:** 2023-01-25

**Authors:** Nuno Gomes, Matilde Pato, André Ribeiro Lourenço, Nuno Datia

**Affiliations:** 1 ISEL, Lisbon School of Engineering, R. Conselheiro Emídio Navarro 1, 1959-007 Lisboa, Portugal; 2 LASIGE & IBEB, FCUL, Universidade de Lisboa, Campo Grande, 1749-016 Lisboa, Portugal; 3 FIT-ISEL, R. Conselheiro Emídio Navarro 1, 1959-007 Lisboa, Portugal; 4 CardioID Technologies Lda., Rua Conselheiro Emídio Navarro 1, 1959-007 Lisboa, Portugal; 5 NOVA LINCS, NOVA School of Science and Technology, 2829-516 Caparica, Portugal

**Keywords:** biosensors, wearables, anxiety, panic disorder, stress, mental health, monitoring, biomarkers in psychiatry, wearable sensor review

## Abstract

Mental illness, whether it is medically diagnosed or undiagnosed, affects a large proportion of the population. It is one of the causes of extensive disability, and f not properly treated, it can lead to severe emotional, behavioral, and physical health problems. In most mental health research studies, the focus is on treatment, but fewer resources are focused on technical solutions to mental health issues. The present paper carried out a systematic review of available literature using PRISMA guidelines to address various monitoring solutions in mental health through the use of wearable sensors. Wearable sensors can offer several advantages over traditional methods of mental health assessment, including convenience, cost-effectiveness, and the ability to capture data in real-world settings. Their ability to collect data related to anxiety and stress levels, as well as panic attacks, is discussed. The available sensors on the market are described, as well as their success in providing data that can be correlated with the aforementioned health issues. The current wearable landscape is quite dynamic, and the current offerings have enough quality to deliver meaningful data targeted for machine learning algorithms. The results indicate that mental health monitoring is feasible.

## 1. Introduction

Technology has been widely adopted in healthcare environments in an effort to improve quality and increase the efficiency of care delivered to patients [1]. This adoption has been gradual, but it accelerated during the recent COVID-19 pandemic event [2]. Wearable sensors have the potential to revolutionize the way we monitor and manage mental health [3,4]. Some examples include:

(1) Tracking physiological parameters: Wearable sensors can continuously track physiological parameters, such as heart rate (HR) and breathing patterns, which can provide valuable insight into an individual’s mental health. For instance, changes in HR may indicate the presence of stress or anxiety [5].
(2) Tracking behavioral parameters: Wearable sensors can track behavioral parameters, such as sleep quality, physical activity, and social interactions, which can provide valuable insight into an individual’s mental health [6,7]. For example, changes in sleep patterns or social interactions may indicate the presence of depression or anxiety.
(3) Providing real-time feedback: Wearable sensors can provide real-time feedback to individuals about their mental health, helping them identify patterns and trends that may indicate the need for intervention [8,9]. As an example, a wearable sensor is capable of alerting an individual to an increase in their HR, which may indicate the presence of stress or anxiety.
(4) Providing personalized interventions: Wearable sensors can be used to deliver personalized interventions based on the data they collect. For example, a wearable sensor that tracks sleep quality may provide individuals with recommendations so they can improve their sleep habits [6]. A wearable sensor that tracks physical activity may provide an individual with personalized exercise recommendations [10].
(5) Enhancing traditional mental health interventions: Wearable sensors can be used to enhance traditional mental health interventions, such as therapy or medication, by providing real-time data that can help inform treatment decisions [11,12].

However, there are several challenges associated with the usage of wearable sensors in mental health. These include issues related to the accuracy and reliability of the data collected, the privacy and security of the data, and the need to develop effective interventions based on the data [13]. Despite these challenges, the potential for wearable sensors to improve mental health outcomes is significant, and interest in this area will likely continue to grow in the coming years. In recent years, there has been more interest in using wearable sensors to monitor mental health [3,11]. This is partly due to the increasing prevalence of mental health issues, such as anxiety and depression, and the need for more effective and accessible interventions, according to a scientific brief released by the World Health Organization (WHO).

Multiple upgrades in a medical setting concerning the presence of technology to facilitate telehealth are also essential for keeping patients out of the hospital. This upgrade has occurred in multiple places, i.e., in the usage of wearables, data visualization [14], the assistance of artificial intelligence (AI), machine learning (ML) in decision-making [15,16], and upgrading an existing electronic medical record (EMR) to state-of-the-art solutions. Monitoring patients has been an essential part of looking after patients. This is because performing multiple examinations on patients is the modus operandito diagnose a patient. In theory, an abundance of readily available non-invasive data about a patient would allow medical professionals to start addressing an issue with previous information and possibly better target a condition with fewer hospital resources.

Remote patient monitoring (RPM) provides a way to collect data from an individual, and can be achieved with invasive sensors, wearables, or questionnaires [17]. Regarding AI and ML, many research projects show that combining AI with human judgment produces the most reliable outcomes; it must be noted that this research is still in its infancy [18]. However, RPM does not address all health issues equally.

This review paper investigates the following significant aspects of mental health monitoring:

(1) Wearable devices for mental health monitoring;
(2) Commercial wearable sensors designed for mental health monitoring;
(3) Value in monitoring stress and anxiety;
(4) Detection and prediction of acute symptoms.

In this paper, we did not consider as valid:

(1) Studies whose primary focus was not on detecting anxiety disorders, such as stress or depression; and
(2) Studies investigating children, since their bodies are still developing, and the data are not inherently applicable to adults.

The remainder of this manuscript is structured as follows. Section 2 discusses the existing survey and review papers in the literature, including their advantages and disadvantages. Section 3 describes the methodology of the search strategy and data collection, inclusion criteria, and exclusion criteria of the selected studies. Then Section 4 describes the brief literature survey about stress detection using wearable sensors and ML techniques, some of the limitations associated are discussed. In Section 5, a conclusive summary of state-of-the-art technologies is given. The conclusions section also indicates new potential directions that we believe will be the focus of wearable sensors to monitor panic disorder (PD) research in the scientific community (in the near future).

## 2. Literature Review

There are some surveys available in this area of RPM. A recent survey from [19], covering 46 studies, concluded that sensors used in the last decade that addressed mental illness problems, in particular those embedded in smartphones, were generally not the most appropriate fits for mental health applications. Vegesna et al. [17] performed a literature review of 62 studies, focusing on RPM via non-invasive digital technologies for remote patient monitoring. Conclusions are drawn based on the authors’ evaluations of the outcomes, which were either positive, negative, or neutral, depending on the health issues they addressed [17]. As stated by the results of their evaluations, wearables had a 50% positive outcome with the rest of the outcomes being neutral; smartphones improved the outcomes of the studies in 58% of the cases, while having no negative impacts in the remainder of the cases (neutral). Study designs included randomized controlled trials (RCTs), observational studies, and systematic reviews published from 1 January 2005 to 15 September 2015.

Kulkarni et al. [20] reviewed several papers on smartphone sensing for health between 2017 and 2022. The review covered a broad range of projects employing different approaches and tools for collecting and analyzing health data. The review identified 71 empirical studies, with mental health conditions being the most prevalent (59%). Additionally, it has reinforced the need for standardization. The review also highlighted the potential of collaboration between human activity and clinical communities in order to improve the efficiency of the project. This will allow researchers to make more effective decisions when it comes to developing new applications that use smartphone sensing. We also provide an overview of the various advancements that have occurred in the field of smartphone sensing.

According to the authors in [21], mobile health (mHealth) devices have the potential to decrease the costs of both clinical research and healthcare just as technological advancements have done in virtually all industries except healthcare. mHealth refers to the integration of mobile telecommunications and multimedia technologies into increasingly mobile and wireless healthcare delivery systems. In addition to supporting diagnostic procedures, mHealth apps can be used by doctors and patients to guide treatment recommendations and educate about diseases. Within the past decade, the creation of commercially available smart devices and wearable technologies to monitor health has grown exponentially [22], and with it, the use cases for wearables have also increased. This paper provides a review on the applicability of wearables in the setting of monitoring anxiety disorders and panic attack (PA), while addressing any secondary measurements that could relate to this, such as the association between anxiety and stress [23].

## 3. Methodology

In this section, we outline how we will conduct the present survey, including the criteria for selecting the relevant papers.

### 3.1. Eligibility Criteria

The current paper defines a set of search terms (STs) deemed relevant and generic. Once the ST is defined, the search algorithm is constructed using the logical operators AND and OR to combine them. Our search criteria identified three distinct categories in which to seek papers. The first looks for all words associated with the sensor, such as “wearable sensors” or “sensor devices”. The second is based on terms related to diseases, for instance,

- “anxiety disorder”,
- “panic disorder”, or
- “panic attack”.

Finally, the third ST is used to find prediction methods that fit the AI field: “machine learning” and “prediction”.

The inclusion criteria include:

(1) Common wrist wearable sensors, such as photoplethysmography (PPG), electrocardiogram (ECG), accelerometer (ACC), electrodermal activity (EDA), breath rate (BR), and skin temperature (TMP);
(2) Metrics related to mental disorders;
(3) Methodology and result objectives (clear);
(4) User testing on people over 18 years old; and
(5) ML processing.

### 3.2. Data Collection Process

Based on the PRISMA (Preferred Reporting Items for Systematic Reviews and Meta-Analyses) guidelines [24], the flow diagram provides detailed information about the study selection process; only papers identifying mental health with wearables were considered.

An electronic journal database search was conducted to provide a comprehensive list of scientific papers:

(1) Google Scholar (n=3950),
(2) Springer Link (n=185),
(3) IEEEXplore (n=30),
(4) MDPI (n=65), and
(5) ACM Digital Library (n=320).

Although we filtered by “Title” and “Abstract”, we found that some systems, i.e., journal database, also included the “Keywords” of the manuscripts. All documents between 2015 and November 2022 were included in the search. The results were analyzed to create a list of potential papers that were peer-reviewed, well-documented, well-written, in English, and provided at least 10 references. Technical reports, surveys, master’s, and Ph.D. theses were removed. This study examined only the top 100 publications on Google Scholar, sorted by their five-year H-index metrics. Then, the abstracts were checked to see if they met the inclusion criteria.

**Figure 1:**
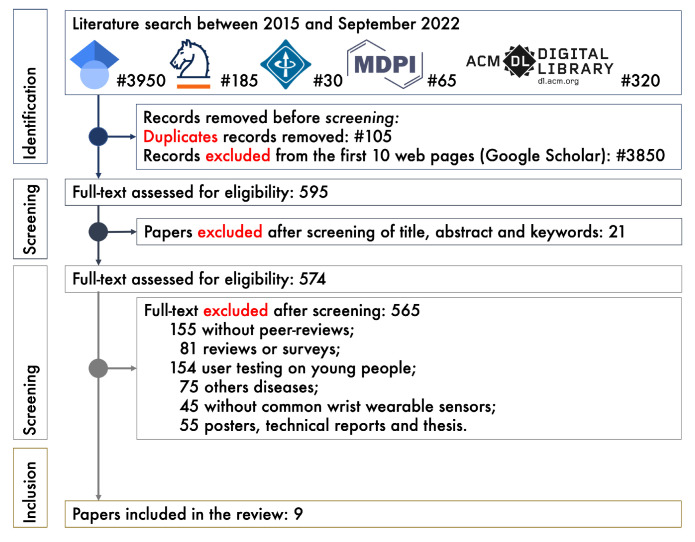
PRISMA flow diagram for the systematic review.

Figure 1 details the methodology used to select the final papers to be included in the systematic review. When screening papers, the inclusion and exclusion criteria described above were used to select and eliminate them. Only the top 595 publications were considered to be eligible. In addition, 21 papers were excluded after reading their abstracts as they were outside the scope of the review. Following a thorough reading of the entire content, 565 papers were removed from the remaining 574 papers (inclusion). Thus, 9 papers remained for study in this systematic review.

## 4. Results and Discussion

This paper was devised as a viability analysis for the detection of mental health conditions using wearable devices. To increase the success probability of future RPM projects targeting mental health issues, we analyzed the current state of wearable usage in the population in Section 4.1. In addition, current sensors used in consumer wearables are examined and presented in Section 4.2. In Section 4.3, we discuss the potential value in monitoring mental health statistics. Various complementary monitoring methods are also analyzed. Lastly, Section 4.4 presents the studies that best resemble systems able to present mental health metrics and predict acute symptoms related to those mental health disorders. This deeper analysis was performed with the intent of developing a future project focused on monitoring and providing help to mental health disorders using wearable sensors as the data capturing method.

Monitoring of PAs is not a novel topic, but the usage of consumer wearables in this field is (having started around the 2010s). Due to the presented arguments, and a certain distrust in the quality of these wearables as data sources, few studies have examined the feasibility of using them to monitor and possibly apply biofeedback to anxiety disorder patients using wearables. We present Table 1 with a review of the few studies regarding the measurement of panic scenarios, with two studies being particularly deeply analyzed. Results are listed in chronological order. It is observable that the simultaneous use of questionnaires and physiological measures, particularly over the past few years, add value to improve the diagnosis. Another relevant datum involves the data recorded with the insertion of EDA and sleep tracking.

Regarding the detection of PD, Elgendi et al. [33] presented mixed results based on experiments using ECG signals. While some cases presented a correlation between panic attack (PA) patterns and ECG patterns, other cases seemed to be uncorrelated. One key finding is that the majority of the test subjects suffered from co-morbid depression (up to 60%), making it difficult to distinguish between subjects with and without psychiatric co-morbidity. An equally significant factor is that none of the studies analyzed revealed the electrodes or wearable placements, which might have impacted some study outcomes. As a result of the positive study results, the HRV feature of high frequencies seemed to be the most reliable metric for panic prediction.

### 4.1. Wearables for Mental Health Monitoring

Monitoring patient data while they live their daily lives is not a novel concept in the medical community. Some devices are already widely used while keeping that premise, such as the 24-h Holter monitor for ECG measurements [34,35]. The future seems to be heading in the way of applying more digital technologies in the medical field, as they are continually being adopted as an additional method for healthcare systems to increase patient contact and augment the practice of preventive medicine [17]. The usage of wearables had no negative impact on the outcome of the work [22,35,36,37,38]. In most of these cases, wearables are used for the continuous monitoring of patients, and the benefits far outweigh any drawbacks. Considering the scenarios presented, it is about obtaining a good amount of data to analyze or having a small dataset, and in this case, wearables are a good way to obtain good-sized datasets that are above average quality. The common usage of wearable devices already makes them great candidates, given that in 2018, one in five individuals in the United States owned a wearable device, most of which are activity trackers. In combination with value-based healthcare systems through telehealth, wearable devices can enable the monitoring of at-risk patients, intervening diseases at an earlier stage, and reducing healthcare expenditures by means of the prediction and prevention of disease [36].

Citing Steinhubl et al. [21], wearable biometric sensors allow for unobtrusive, passive, and continuous monitoring, and one of the key characteristics is their ability to seamlessly track and transfer all biometric data into an actionable and informative user interface that can be shared with healthcare providers and researchers. In the end, the amount of data on record from a single patient, allows healthcare professionals to address their patients according to individualized (that is, precision or personalized) medicine.

Some wearables are already in the race to be used as high-quality data capturers. For instance an FDA-cleared, wearable–multi-parameter vital sign monitoring device records and transmits

(1) ECG;
(2) HR;
(3) Respiratory inductance plethysmography;
(4) Calorific expenditure;
(5) Posture/activity;
(6) Skin/core temperature; and
(7) Oxygen consumption [36].

Experimental studies to monitor physiological signals and ECG for detecting arrhythmia have been successfully carried out using wearable sensors this way, according to results on wearable sensors [36]. A number of studies [6,39] showed the potential to accurately classify sleep apnea data, based on low-cost and easy-to-use sensors. A proof-of-concept of a radar-based vital sign detection system is presented to monitor heart rate variability (HRV) [40].

Wearable sensors can be used to target mental health issues in a few forms. Starting with the most direct form, targeting symptoms, mental health disorders contain common somatic symptoms among them, such as:

(1) Palpitations (increased or irregular heart rate (HR));
(2) Sweating;
(3) Tremors;
(4) Muscle tension;
(5) Dizziness;
(6) Nausea; and
(7) Hyperventilation [41,42,43].

Using sensors, we can specifically target the occurrence of symptoms, such as using HR to target palpitations, EDA to target muscle tension, sweating and tremors, TMP to target muscle tensions, and respiratory rate (RR) to target hyperventilation.

In an indirect form of targeting mental health disorder activity, the activity of the nervous system can be measured. Some mental health disorders imply heavier sympathetic nervous system (SNS) activity since these states are responses in “fight or flight” situations, when a person is presented with physical danger or mental stress, and can turn off non-essential functions, such as digestion. The activity of SNS happens in a balance with the parasympathetic nervous system (PNS), meaning an increase in one’s activity implies a decrease in the other’s activity.

Regarding sensors that can target these indirect indicators, we can use EDA. This sensor mainly targets the production of sweat by the human body, which is by itself a non-voluntary action, pursued by the SNS. It is one of the few non-invasive measures of sympathetic arousal. Another important vital signal that is controlled by both the SNS and PNS during their different control states is the HR, which can be analyzed to track specifically signatures of SNS or PNS activity.

For reliable, continuous, and personal monitoring, the measurements must be made outside the clinical environment using semi-validated, wearable devices that are tolerated by people [22]. The non-invasiveness of the sensors must especially be considered since the constant reminder of being analyzed might change a user’s behavior and data, and the discomfort of usage might hinder long-term adherence to any given project. According to some authors [35,37,38], the optimal sensor location should ensure the accuracy of sensors and minimize visibility. They are unanimous and it is concluded that patients have higher preference and compliance for devices placed on the wrist, as such, devices can often be perceived to be clothing accessories. A project meant for the monitoring of mental health data heavily relies on having good adherence by the users (since without continuous data from big swaths of time, it is not possible to infer the baseline and, consequently, detect erratic data values).

### 4.2. Off-the-Shelf Wearables for Mental Health

Wearables can take a wide range of metrics. In various settings, multiple wearable sensors have been tested, and research projects have been conducted. In this section, we present a variety of sensors used in the current wearable market. Details of the commercially-available off-the-shelf wearables are compiled in Table 2. From this, the devices down-selected for consideration are:

(1) EQ02 LifeMonitor belt (Equivital, Cambridge, UK);
(2) BioPatch^™^ HP (Zephyr Technology, Annapolis, MD, USA);
(3) Hexoskin Smart Garment (Carré Technologies Inc., Montreal, QC, Canada).

The watches/devices (wristwatches) are:

(1) Empatica embracePlus and empaticaCARE (Empatica Inc., Boston, MA, USA);
(2) Inspire 3 and Sense 2 (Fitbit, San Francisco, CA, USA);
(3) Venu^®^ Sq2 (Garmin, Ltd., Schaffhausen, Switzerland); and
(4) Mi Band (Xiaomi Corp., Beijing, China).

Most of these sensors are relatively common and easy to find in a pre-built wearable system, sometimes even bundled in groups of 2 or 3, which makes these devices interesting from a physiological data capture perspective [44].

The EQ02 LifeMonitor https://equivital.com/, accessed on 14 November 2022 belt (Equivital, Cambridge, UK) is suitable for multi-parametric ambulatory health monitoring since it correlates well with standard laboratory devices for all physiological measures. The aforementioned sentence is validated in [45]. The BioPatch^™^ HP https://www.zephyranywhere.com/, accessed on 14 November 2022 (Zephyr Technology, Annapolis, USA ) is an FDA-approved device and was used as the gold standard to compare data obtained from the other devices regarding their accuracy on HRV. Cruz et al. [25] applied this wearable in detection of the attacks, which sampled HR, TMP, BR, and HRV via the usage of ECG electrodes. Hexoskin Smart Garment https://www.hexoskin.com/, accessed on 14 November 2022 (Carré Technologies, Inc., Montreal, Canada ) consists of sensorized undergarments and data loggers. The validity of the Hexoskin measurement of HR, respiratory rate (RR) was evaluated [46,47]; the results indicated that HR and RR are strongly correlated to the reference standard. Empatica https://www.empatica.com/, accessed on 14 November 2022 (Empatica Inc., Massachusetts, USA) developed two CE certified smartwatches for continuous health monitoring, embracePlus, and empaticaCARE. The digital biomarkers were designed to track patients with epilepsy, physical well-being, mental health, and respiratory infections. To date, no white papers or validation studies exist for this device [35]. Fitbit wearables https://www.fitbit.com/, accessed on 14 November 2022 (Fitbit, San Francisco, USA), Inspire 3 and Sense 2, are also commonly used in research, providing reliable results and consistent readings, with the downfall of being more expensive than most consumer products [10,48]. Among all brands studied, Fitbit is the most popular [49]. There are several Garmin https://www.garmin.com/, accessed on 14 November 2022 (Garmin Ltd., Schaffhausen, Switzerland) watches available on the market. During swimming activities, Cosoli et al. [50] evaluated the accuracy and precision of wrist-worn (Polar Vantage V2 and Garmin Venu Sq) and chest strap (Polar H10 cardiac belt) commercial HR sensors. In a macro view, there are not enough studies reporting analysis performances [50]. Ideally, cheap and accessible wearables, such as the Mi Band https://www.mi.com/global/, accessed on 14 November 2022 (Xiaomi Corp., Beijing, China) line, could be the perfect product to develop the idea, given the ubiquity among wearable users, but their consistency on readings needs more research [51,52].

### 4.3. Value in Monitoring Stress and Anxiety

After the aforementioned pandemic, there is a push toward better mental healthcare and recognition. This section will focus on the work that provides successful results with wearable compatible sensors in measuring stress, anxiety, or PAs. This involves the use of sensor data and ML to differentiate each metric.

Considering the focus of this paper, a comprehensive collection of sensor information to measure each metric will be of significant value. This will allow for better choices of future sensor combinations. The pioneers of this field are Healey et al. [53], who showed that, in 2005, stress can be detected using physiological sensors. In the mentioned paper, the objective was to distinguish between three base levels of stress in drivers (low, medium, and high), with an accuracy rate better than 97%. It is the stress with a negative bias (namely distress) that is addressed in the study. Four types of physiological sensors are used during the experiment:

(1) ECG;
(2) electromyogram (EMG);
(3) Skin conductivity (also known as EDA or galvanic skin response (GSR)); and
(4) Respiration (through chest cavity expansion).

Their algorithm included the mean and variance of the EMG taken by the hand, as well as the respiration and the mean of the tachometer HR over one-second intervals throughout the drive. Using 100- to 300-second windows to confirm how closely the sensor features vary with stress metrics, HR and skin conductivity are positively correlated with stress. The authors found that 97% of the time, they could accurately determine stress levels by measuring heart rate and skin conductivity at intervals of five minutes.

Later, Gjoreski et al. [54] presented a method that unobtrusively monitors psychological stress in real life, using a wrist device. The work consists of detecting stress in both constrained and unconstrained environments. Since stress is a response to physiological, behavioral, and affective aspects, the sensors can only provide direct physiological data. The wristband can provide, among others, EDA and HR. The data are then used to create several ML models using different learning algorithms (e.g., support vector machine (SVM)), with a multitude of techniques and variations. The conclusion is that contextual information provides key clues in stress detection. The most accurate method is able to detect (recalls) 70% of stress events with a precision of 95%. However, context depends on a high-quality activity detector.

The authors of [55] monitored 17 subjects daily in a laboratory setting and the stress status was identified (stressed or not-stressed). The paper focused on the usage of three sensors, PPG, ECG, and GSR, also known as EDA. The study proved to have 94.6% accuracy in detecting stress levels in a lab setting, but this dropped to 81.82% in an everyday setting. According to the study, the GSR + ECG group had the maximum everyday accuracy, with 90.91% accuracy in the everyday setting [55]. Another finding is that the sensors from the wearables tended to perform worse on an everyday setting since noise becomes a issue when it comes to measuring data in a non-lab scenario. While this outcome is not unexpected, it is important to notice the difference.

For an overlook of the current situation in measuring stress, we can see value in all of the presented sensors. While ECG and PPG are relatively recent widespread sensors; they are also promising, as HRV is identified as the most useful physiological metric for the detection of stress and anxiety [22]. It was also observed that HR and GSR are the most regularly used sensory signals because they gave the most promising results and high accuracy for detecting stress and its levels [4].

Regarding anxiety, ecological momentary assessment (EMA), which stands for continuous measurement, has the potential to shift the anxiety and panic medical area perspective toward a “precision psychiatry” approach in which intensive time series data are used to tailor the treatment of anxiety disorders to individual patients [56]. The increase in incidence of anxiety disorders worldwide has motivated researchers to develop new and improved technologies to promote well-being and reduce related morbidity, mortality, and healthcare costs [33].

Continuous monitoring of this kind of patient is seen as a sound idea due to the ease of knowing and adapting to each patient’s specific constellation of symptoms, and understanding the dynamics of the symptoms, as well as the relationships among them [56].

Measuring anxiety and stress overlaps in a lot of ways, and this hinders the distinction between which sensors to use for each subject, or if a sensor can even be tasked with the objective of distinguishing them. The overlap between depression, anxiety, and stress is in part motivated by the fact that they share many risk factors and symptoms. Nevertheless, the reason for the association between these psychological syndromes is yet to be established [57]. For all the monitoring, context is likely to be important [54] since it allows performing a better evaluation of the data, and questionnaires can fill the gap as presented in [18]. If questionnaires are to be used, they will need to be used sparingly, as they can cause questionnaire fatigue, undermining adherence to the project, which adds further importance to context detection on the wearable itself.

Chung et al. [29] presented positive results on how to reduce symptoms of anxiety with a HRV biofeedback wearable. Over the course of eight weeks, the patients performed a GAD-2 questionnaire, which stands for generalized anxiety disorder scale with 2 questions to measure symptoms of anxiety, reaching a maximum total score of 6. As the study progressed, the patient scores dropped three points (less is better). The results suggested that HRV biofeedback through a discrete wearable device combined with a remote stress management program may be effective at reducing depression and anxiety symptoms. The authors chose an ECG sensor, in spite of the PPG, since early studies (e.g., [58]) suggest the first sensor to be more reliable, particularly when movement is introduced. The feedback portion is delivered via the wearable on the chest, taking the form of vibrations so the users can match their breathing patterns.

According to a 2021 literature review by Hickey et al. [22], cardiac activity was the only objectively measurable physiological parameter of anxiety in the literature. However, respiratory patterns have been reported to clearly indicate emotional stress. Moreover, the usefulness of ECG in monitoring anxiety is highlighted in that review. The presented results call into question the ongoing challenge of validating EDA and ACC. The first one because it does not seem to perform accurate measurements on wearable devices, due to noise on the readings. Regarding the measurement of anxiety, there is special importance in alterations in HR, RR, and EDA since they mirror the function of the SNS [22], which is closely tied to anxiety activity; thus, the measurements of these signals have heightened values to classify a state.

### 4.4. Detection and Prediction of Acute Symptoms

Systems using ML and AI are not new. They have been around since the 1950s, and there have been several breakthroughs, followed by some stagnation—the so-called AI winters [59]. Several factors may influence those cycles, including limitations on the computational power of hardware and software. Nowadays, AI in general, and ML in particular, are expanding fields of research [60].

Recent developments have led to an approximation between ML and medical data. In the physiological area, related to stress, Aqajari et al. [61] developed a Python Toolkit to preprocess and classify stress responses from EDA. The presented classifiers achieved accuracy between 80% and 97%, identifying stress responses from EDA data from different sources of data.

Physiological data can be assessed by comparing them to standard reference intervals. It is the way it has mostly been done until now, and it still is in some areas. For instance, when a person takes a blood sample for analysis, the values are considered healthy if they fit into a predefined reference interval. The approach fits the majority of people, but there are still healthy people who have different reference values. Therefore, there is space for a personalized approach. This approach starts with common intervals and then takes into account individual data and context from the patient to deliver a personalized approach.

With continuous data from the patients, via the usage of wearables, it is possible to have a steady source of baseline data. Applying a form of ML in this case, to try and detect anomalous data, and then reacting to that information, brings more value to a GAD and PD support system. The research is still relatively new in the area, so it is difficult to delve deep into it, but detecting stress has been researched already. In a survey from 2021, Gedam and Paul [4] examined if the data from multiple sensors in wearables could be used to apply ML techniques and recognize stress in the presence of stressors. It was observed that HR and GSR are the most regularly used sensory signals because they gave the most promising and highly accurate results for detecting levels of stress [4]. The most used classifiers in this case are K-nearest neighbor (KNN), random forest (RF), and support vector machine (SVM). Moreover, there is considerably better accuracy when using data from more than one biosensor. The majority of the accuracies in stress classification presented are higher than 90%, which makes this method a viable approach to detecting stress [4].

On the subject of detection and prediction of PD, Cruz et al. [25] is the most appropriate subject for comparison. The collected data are used to build feature vectors, and anomaly detection algorithms are used to distinguish between pre-panic and non-panic intervals. Regarding the usage of ML in the context of physiological data, the literature is quite recent, and almost non-existent when taking into account the diseases and mental health monitoring using wearables. Nevertheless, we found usage of the generalized linear mode (GLM) [28], KNN, SVM [30,54,62,63], RF [30,32,62,63], logistic regression (LR) [30], extreme gradient boosting (XGBoost) [32], linear discriminant analysis (LDA) [32], AdaBoost [32,62], decision trees (DTs) [32,62], Bayesian networks (BNs) [62], and artificial neural networks (ANNs) [62].

The research focused on various single points that can be applied to mental health monitoring solutions. However, these single points are not necessarily presented in a cohesive manner. Each factor seems to have ground to stand on its own, namely, the quality of the currently wearable sensors, the analysis of data from said sensors, and the classification. We also want to understand if a system incorporating all of the presented arguments and presenting a user-facing interface would add more value regarding mental health monitoring than all of those parts combined.

## 5. Conclusions

In this survey, we revised the state-of-the-art research that addressed mental health issues using wearable sensors. The majority of the studies we found were based on studies on children under 18. Therefore, we had to discard many of the papers found. Despite the limited number of works available, indicating a domain that needs more attention, the available results are promising. Based on the results of this study, it seems that there is a place for mental health monitoring using wearables as capturing data devices. Therefore, there is a vast array of off-the-shelf wearable devices available that measure physiological data, including HR, sleep, and breathing patterns, which can potentially be used to identify and monitor PAs in real-time. These devices can be worn on the wrist, chest, or other parts of the body, and often sync with a smartphone or another device to provide real-time data and analysis. Many of these wearables use ML algorithms to analyze and interpret the data they collect, and can provide users with insights and feedback about their health and wellness. ML is relatively new in the context of physiological data, and almost nonexistent when it comes to diseases and wearable health monitoring. The use of multiple complete ML-trained classifiers on a wrist-wearable is becoming possible thanks to advances in the computational power of wearable devices. High-quality biosensors are becoming available in a variety of forms, most of them as less intrusive wearables, making it possible to achieve high-quality biosignals. Combining this technology with a deeper understanding of the contextual factors that trigger mental health issues will help us to recognize possible mental health events in time to better address them. It should be as simple as looking at your wrist and realizing it is time to take care of yourself. The use of consumer wearables for the monitoring of PA is an area of active research, and it is likely that new developments in this field will continue to emerge in coming years.

There are several potential directions for future work in this field. One potential direction is the integration of wearable devices with other technologies, such as virtual reality or telehealth, to provide more comprehensive support for individuals with PD. This could include the use of virtual reality-based therapy or other interventions to help individuals cope with PAs and manage their conditions more effectively. Finally, further research is needed to understand the long-term effectiveness of wearable devices regarding the monitoring and management of PD, and to identify any potential risks or unintended consequences of their use.

## Figures and Tables

**Table 1:** An overview of all papers that use wearable devices to perform anxiety-related measurements. The first column shows the papers’ authors. The second column lists physiological data. The table foot list the wearable sensors, the questionnaires, and the setup (if any). In the sixth column, the objective is presented, followed by the results of each objective. The abbreviations used in the table can be found at the end of the table.

Paper	Data	Devices	Questionnaires	Setup	Objective	Results
Cruz et al. [25]	TMP, BR, HRV & HR	Zephyr Biopatch & Smartphone.	N.A.	Smartphone, wearable & data center.	Mobile and wearable system to detect PAs as they occur, with the option to self-report.	PAs can be detected as they occur, via the used measurements. The physiological signs from a PA can manifest until an hour before the attack.
Rubin et al. [26]	HRV & HR	From a dataset, multiple devices.	N.A.	N.A.	Create models to distinguish between panic states, as PA, pre-PA, and non panic.	Combining time, frequency, and nonlinear domain parameters, models can achieve > 90% accuracy when distinguishing between panic states in physiologic time-series data.
McGinnis et al. [12]	HR, HRV & BR	Smartphone Camera	One, proprietary.	Mobile and wearable system to detect PAs.	Provides in vivo biofeedback therapy for PAs, shown on the smartphone screen.	The modality of measurement is feasible with a smartphone camera. There is an apparent placebo effect with the measurement procedure since it acts to stop the attack.
Puli et al. [27]	ACC & HR	Shimmer 2r.	N.A.	Wearable used to capture data and models applied post-capture.	Proposing and testing an approach for real-time detection of anxiety arousal in autism spectrum disorder patients, taking movement into account.	Evaluation using data from afflicted children showed an overall arousal detection accuracy of 93%. This method showed promising results regarding the avoidance of false positives.
Perpetuini et al. [28]	BP & HR	Custom device using PPG.	STAI.	Custom-wearable used to capture data to be analyzed post-recording.	Possibility of using a non-invasive technique (PPG) to encode information about emotional conditions, namely estimating the state anxiety of healthy participants.	The results proved that data from PPG is a good indicator of state anxiety. The results provided a correlation of 81% between the ML algorithm and state anxiety.
Chung et al. [29]	HRV & HR	Lief Smart Patch.	GAD-2 & PHQ-2.	Device that captures data and provides biofeedback.	The feasibility of using this system to decrease overall levels of anxiety disorders, including PD in patients.	This research involves the self-reported anxiety of 3 points (range 0–8) and 1 point in depression (0–8). This paper was conducted over 56 days.
Nath et al. [30]	EDA, HR & BP	Non-specified wearable wristband.	STAI.	Wearable used to capture data to be analyzed post-recording.	The viability of anxiety detection in older adults, using low-cost wearables to capture physiological data.	An accuracy of 92% was accomplished using EDA features and context, without context, this accuracy dropped to 89%. This work showed potential in using EDA to detect anxious states.
Shaukat-Jali et al. [31]	HR, EDA & TMP	E4 Empatica.	LSAS-SR.	Wearable used to capture data and models applied post-capture.	The aim of the paper was to detect if subclinical social anxiety could be detected using physiological data obtained from wearable sensors.	The models presented accuracy values between 97.54% and 99.48% when differentiating between baseline and socially anxious states. EDA is identified as the most effective feature to differentiate between states.
Tsai et al. [32]	Sleep rate, HR & Activity	Garmin Vivosmart 4.	BDI, BAI, STAI-S, STAI-T, and PDSS-SR.	Wearable used to capture data and mobile app to collect data.	Viability of predicting PAs up to seven days in advance.	Up to seven days of prediction; the best presented accuracy was 81.3% using physiological data and questionnaires.

**Sensors**: accelerometer (ACC), breath rate (BR), blood pressure (BP), electrodermal activity (EDA), heart rate (HR), heart rate variability (HRV), photoplethysmography (PPG), and skin temperature (TMP). **Questionnaires**: generalized anxiety disorder scale, 2 questions (GAD-2), patient health questionnaire, 2 questions (PHQ-2), post-traumatic adjustment scale (PAS), agoraphobic cognition questionnaire (ACQ), body sensation questionnaire (BSQ), Hamilton anxiety rating scale (HAM-A), mobility inventory (MI), Hamilton anxiety rating scale, reviewed (SIGH-A), Centre for epidemiological studies depression scale(CES-D), Liebowitz social anxiety scale (LSAS-SR), Beck depression inventory (BDI), Beck anxiety inventory (BAI), state-trait anxiety inventory state anxiety (STAI), state-trait anxiety inventory trait anxiety (STAI-T), panic disorder severity scale self-report (PDSS-SR), German adaptation of CES-D (ADS), and 12-item short-form health survey (SF-12). **Terms**: cognitive behavioral therapy (CBT), panic attack (PA), panic disorder (PD). Others: Not available (N.A.).

**Table 2:** Commercially available off-the-shelf wearable sensors.

Sensors	EQ02 LifeMonitor	BioPatch HP	Hexoskin Smart Garment	Embrace Plus & Empatica CARE	Inspire 3 & Sense 2 (Fitbit)	VenuSq2 (Garmin)	Mi Band (Xiaomi)
ACC	✓	✓	✓	✓		✓	✓
Activity	✓	✓	✓			✓	
BP	✓						
ECG	✓	✓	✓		✓		
EDA				✓			
HR	✓	✓	✓		✓	✓	✓
HRV	✓	✓			✓	✓	
Body position/movement	✓	✓	✓	✓	✓	✓	
PPG			✓	✓			✓
RR	✓	✓	✓		✓	✓	
Sleep tracking	✓		✓	✓	✓	✓	✓
TMP	✓		✓	✓	✓	✓	
SpO2		✓		✓	✓	✓	✓

**Sensors**: accelerometer (ACC), blood pressure (BP), electrocardiogram (ECG), electrodermal activity (EDA), heart rate (HR), heart rate variability (HRV), photoplethysmography (PPG), respiratory rate (RR), skin temperature (TMP), and oxygen blood saturation (SpO2).

## Abbreviations

The following abbreviations are used in this manuscript:

ACC	Accelerometer
ANN	Artificial Neural Network
AI	Artificial Intelligence
BN	Bayesian Network
BP	Blood Pressure
BR	Breath Rate
DR	Decision Trees
ECG	Electrocardiogram
EDA	Electrodermal Activity
EEG	Electroencephalogram
EMA	Ecological Momentary Assessment
EMG	Electromyogram
EMR	Electronic Medical Record
GAD	Generalized Anxiety Disorder
GLM	Generalized Linear Model
GSR	Galvanic Skin Response
HR	Heart Rate
HRV	Heart Rate Variability
IBI	Interbeat Interval
KNN	K-Nearest Neighbor
LDA	Linear Discriminant Analysis
LR	Logistic Regression
ML	Machine Learning
PA	Panic Attack
PD	Panic Disorder
PNS	Parasympathetic Nervous System
PPG	Photoplethysmography
RF	Random Forest
RPM	Remote Patient Monitoring
RR	Respiratory Rate
SNS	Sympathetic Nervous System
SpO2	Oxygen Blood Saturation
SVM	Support Vector Machine
TMP	Skin temperature
XGBoost	Extreme Gradient Boosting
